# Supplementary material for: The binding of Borealin to microtubules underlies a tension independent kinetochore-microtubule error correction pathway
Source: Nat Commun. 2019 Feb 8;10:682. doi: 10.1038/s41467-019-08418-4 (PMC6368601; doi:10.1038/s41467-019-08418-4)
Supplement: Supplementary file 1 — Supplementary Information [file 41467_2019_8418_MOESM1_ESM.pdf]

Supplementary Information

The binding of Borealin to microtubules underlies a tension independent kinetochore-microtubule error correction pathway

(Trivedi et. al.)

Supplementary Table 1: PCR primer sequences for site directed mutagenesis

Mutagenesis primers	Sequence (5' to 3')	Purpose
Bordel2-20fw	TAAGAAGGAGATATACCATGCTCGCCTCCTTTCTGAAAG	Deletion of 2-20 amino acid
Bordel2-20rv	CTTTCAGAAAGGAGGCGAGCATGGTATATCTCCTTCTTA	Deletion of 2-20 amino acid
BorMTBMfw	AGGAGGCGAGCTCCTCCCTCTCTAAGGAGTTGGTCTTGGCCACCC	Mutating amino acids 17,19,20 to E's
BorMTBMrv	GGGTGGCCAAGACCAACTCCTTAGAGAGGGAGGAGCTCGCCTCCT	Mutating amino acids 17,19,20 to E's
BorT230Efw	GCAAAGAGATCTTCCTCGAGGTGCCAGTGGGCGGCGG	Mutating T230 to E
BorT230Erv	CCGCCGCCCACTGGCACCTCGAGGAAGATCTCTTTGC	Mutating T230 to E

Supplementary Table 2: PCR primer sequences for cloning

Cloning primers	Sequence (5' to 3')	Purpose
pET28aINCENP1-58fw	GCCGCGCGGCAGCCATATGGGGACGACG GCCCCAGG	Cloning INCENP in pET28a
pET28aINCENP1-58rv	GCTCGAATTCGGATCTCATTTGGGCATCAG CTCTGGCT	Cloning INCENP in pET28a
pET28aBorflfw	AAGAAGGAGATATACCATGGCTCCTAGGAA GGGCAG	Cloning Borealin in pET28a
pET28aBorflrv	GACGGAGCTCGAATTTCATTTGTGGGTCCG TATGC	Cloning survivin in pET28a
pET28aSurvivinflfw	AAGAAGGAGATATACCATGGGTGCCCCGA CGTTGCC	Cloning survivin in pET28a
pET28aSurvivinflrv	GACGGAGCTCGAATTTCAATCCATGGCAGC CAGCT	Cloning survivin in pET28a
pET28atricisborfw	TAAGCAGAGCTCTAATTTTGTTTAACTTTAA G	Cloning Borealin in pET28a INCENP
pET28atricisborrv	TGCTTAAAGCTTTCATTTGTGGGTCCGTAT GC	Cloning Borealin in pET28a INCENP
pET28atricissurvivinfw	TAATCAGCGGCCGCTAATTTTGTTTAACTTT AAG	Cloning survivin in pET28a INCENP, Borealin
pET28atricissurvivinrv	TGATTACTCGAGTCAATCCATGGCAGCCAG CT	Cloning survivin in pET28a INCENP, Borealin
pET28aPRC1fw	TAAGCAGAATTCATGGTGGATCGGTTGGAA GAACTG	Cloning PRC1 MTBD in pET28a
pET28aPRC1rv	TGCTTAGCGGCCGCCCGGTGTATTGGGAG CCAG	Cloning PRC1 MTBD in pET28a
pET28aPRC1Borfw	TAAGCAGCGGCCGCCTCGCCTCCTTTCTG AAAGAC	Cloning Borealin downstream of PRC1 MTBD pET28a

pET28aPRC1Borrv	TGCTTACTCGAGTTATTTGTGGGTCCGTAT GCTGCT	Cloning Borealin downstream of PRC1 MTBD pET28a
Gtwprcborfw	GGGGACAAGTTTGTACAAAAAAGCAGGCTT CATGGTGGATCGGTTGGAAGAAC	Gateway primers to make donor vector for PRC1-Borealin
Gtwprcborrv	GGGGACCACTTTGTACAAGAAAGCTGGGT CTTATTTGTGGGTCCGTATGCTGC	Gateway primers to make donor vector for PRC1-Borealin
pBabemchborbamh1fw	TCTAGGCGCCGGCCGGATCCATGGTGAGC AAGGGCGAGG	Cloning mcherry borealin in pBabe-puro
pBabemchborecor1rv	CTGTGCTGGCGAATTCTCATTTGTGGGTCC GTATGCTG	Cloning mcherry borealin in pBabe-puro
pdnrincenp747-918fw	GGGGACAAGTTTGTACAAAAAAGCAGGCTT CAAGGAGCAGCTGCAGAGG	Cloning INCENP748-918 in pDonr221
pdnrincenp918rv	GGGGACCACTTTGTACAAGAAAGCTGGGT CTCAGTGCTTCTTCAGGCTGT	Cloning INCENP748-918 in pDonr221
CMVmchborfw	GTGGGCTCTATGGTTAGCCCATATATGGAG TTCCG	Cloning CMV-mch-borealin in TO-LAP-CENPB-INCENP747-918-pCDNA5-FRT
CMVmchBorrv	TCCGCCTCAGAAGTTCCATAGAGCCCACC GCAT	Cloning CMV-mch-borealin in TO-LAP-CENPB-INCENP747-918--pCDNA5-FRT
ISBGFPFw	GCCGCGCGGCAGCCATATGGTGAGCAAGG GCGAGGA	Cloning GFP in pET28a ISB tricistronic vector using coldfusion cloning
ISBGFPRv	GGCCGTCGTCCCCATATGCTTGTACAGCTC GTCCATGC	Cloning GFP in pET28a ISB tricistronic vector using coldfusion cloning

Supplementary Table 3: Sequences of siRNA used in this study:

siRNA	Sequence	Publication
siBorealin 3'UTR	AGGUAGAGCUGUCUGUUCAdTdT	[68]
siLuciferase (siLuc)	CGUACGCGGAAUACUUCGAdTdT	[39]
siINCENP 3'UTR	GGCUUGGCCAGGUGUAUAUdTdT	[39]
siBub1	CCCAUUUGCCAGCUCAAGCdTdT	[69]

Supplementary Table 4: Small molecules used in this study:

Small molecules	Concentration / duration
STLC	5uM for 2hr
5-Iodothio	2uM for 30-45'
MG132	10uM
Nocodazole	0.33uM or 3.3uMfor 20-30min

Supplementary Table 5: Antibodies used in the study

Antibody	Concentration	Application	Source animal	Company/source	Item number
pH3S10	1 in 800	IF	Rb	EMD Millipore	06-570
pH3T3	1 in 1500	IF	Rb	EMD Millipore	07-424
pH2a T120	1 in 1000	IF	Rb	Active motif	61195
Sgo1	1 in 100	IF	Ms	Abcam	ab58023
Aur-B pT232	1 in 200	IF	Rb	Rockland	600-401-677S
Aurora-B	1 in 250	IF	Ms	BD Biosciences	611083
Borealin	1 in 1000	IF/WB	Rb	Stukenberg lab[35] (986)	
INCENP	1 in 1000	IF	Ms	Abcam	ab23956
ACA	1 in 200	IF	Hu	Antibodies Inc.	15-234-0001
CENPA pS7	1 in 100	IF	Rb	EMD Millipore	07-232
Dsn1 pS109	1 in 1000	IF	Rb	Ian Cheeseman lab[17]	
Knl1 pS60	1 in 2000	IF	Rb	Ian Cheeseman lab[17]	
Hec1 pS44	1 in 1500	IF	Rb	Deluca lab[15]	
Hec1 pS55	1 in 250	IF	Rb	Stukenberg lab (974)	
Hec1 pS69	1 in 1000	IF	Rb	Stukenberg lab (975)	
Hec1 (9G3.23)	1 in 2000	IF	Rb	Genetex	GTX70268
Knl1	1 in 1000	IF	Rb	Arshad Desai lab[67]	
Bub1	1 in 1000	WB	Rb	Genetex	GTX30097
mCherry	1 in 1000	WB	Rb	Genetex	GTX128508
INCENP	1 in 1000	WB	Rb	Sigma	I5283
Survivin	1 in 1000	WB	Rb	cell signaling	2808
Tubulin (Dm1a)	1 in 500	IF/WB	Ms	Sigma	T6199

Supplementary Table 6. Model parameters.

symbol	Description	value	units
Binding constants			
k_{on}^{MT}	Association rate constant for kinase to microtubules	2.9	$\mu M^{-1}\ s^{-1}$
k_{on}^{chrom}	Association rate constant for kinase to chromatin binding sites	2.9	$\mu M^{-1}\ s^{-1}$
k_{off}^{MT}	Dissociation rate constant for kinase from microtubules	0.3	s^{-1}
k_{off}^{chrom}	Dissociation rate constant for kinase from chromatin binding sites	0.014	s^{-1}
Enzymatic constants			
k_f^a	rate constant for the formation of the enzyme-substrate complex of active and partially active kinase molecules in case at least one of the molecules is soluble	0.1	$\mu M^{-1}\ s^{-1}$
k_f^b	rate constant for the formation of the enzyme-substrate complex of active and partially active kinase molecules in case both molecules are chromatin-bound	0.001	$\mu M^{-1}\ s^{-1}$
k_f^T	rate constant for the formation of the enzyme-substrate complex of active and partially active kinase molecules in case both molecules are microtubule-bound	0.001	$\mu M^{-1}\ s^{-1}$
k_r^a	rate constant for the dissociation of the enzyme-substrate complex of active and partially active soluble kinase molecules	5.1	s^{-1}
k_r^b	rate constant for the dissociation of the enzyme-substrate complex of active and partially active chromatin-bound kinase molecules	0.21	s^{-1}
k_r^T	rate constant for the dissociation of the enzyme-substrate complex of active and partially active kinase molecules in case both molecules are microtubule-bound	0.21	s^{-1}
k_{cat}^a	catalytic rate constant for active soluble or chromatin-bound kinase toward all forms of the partially active kinase	2.3	s^{-1}
k_{cat}^{MT}	catalytic rate constant for active microtubule-bound kinase toward all forms of the partially active kinase	4.6	s^{-1}
k_{cis}	rate constant for kinase cis-activation, all forms	7.28×10^{-6}	s^{-1}
k_f^p	rate constant for the formation of the enzyme-substrate complex of phosphatase and active kinase, all forms	0.6	$\mu M^{-1}\ s^{-1}$
k_r^p	rate constant for the dissociation of the enzyme-substrate complex of phosphatase and active kinase, all forms	0.09	s^{-1}
k_{cat}^p	catalytic rate constant for phosphatase toward active kinase, all forms	6×10^{-3}	s^{-1}
Other model parameters			
D	diffusion coefficient of soluble kinase	1	$\mu m^2\ s^{-1}$
D_{MT}	diffusion coefficient of microtubule-bound kinase	0.1	$\mu m^2\ s^{-1}$
$PPase_0$	total concentration of phosphatase	0.282	μM
A_{sol}	total concentration of soluble kinase	0.01	μM
MT^{total}	Total concentration of CPC binding sites on microtubules	5	μM
B_0	Peak concentration of CPC binding sites on chromatin	30	μM

Supplementary Figure 1

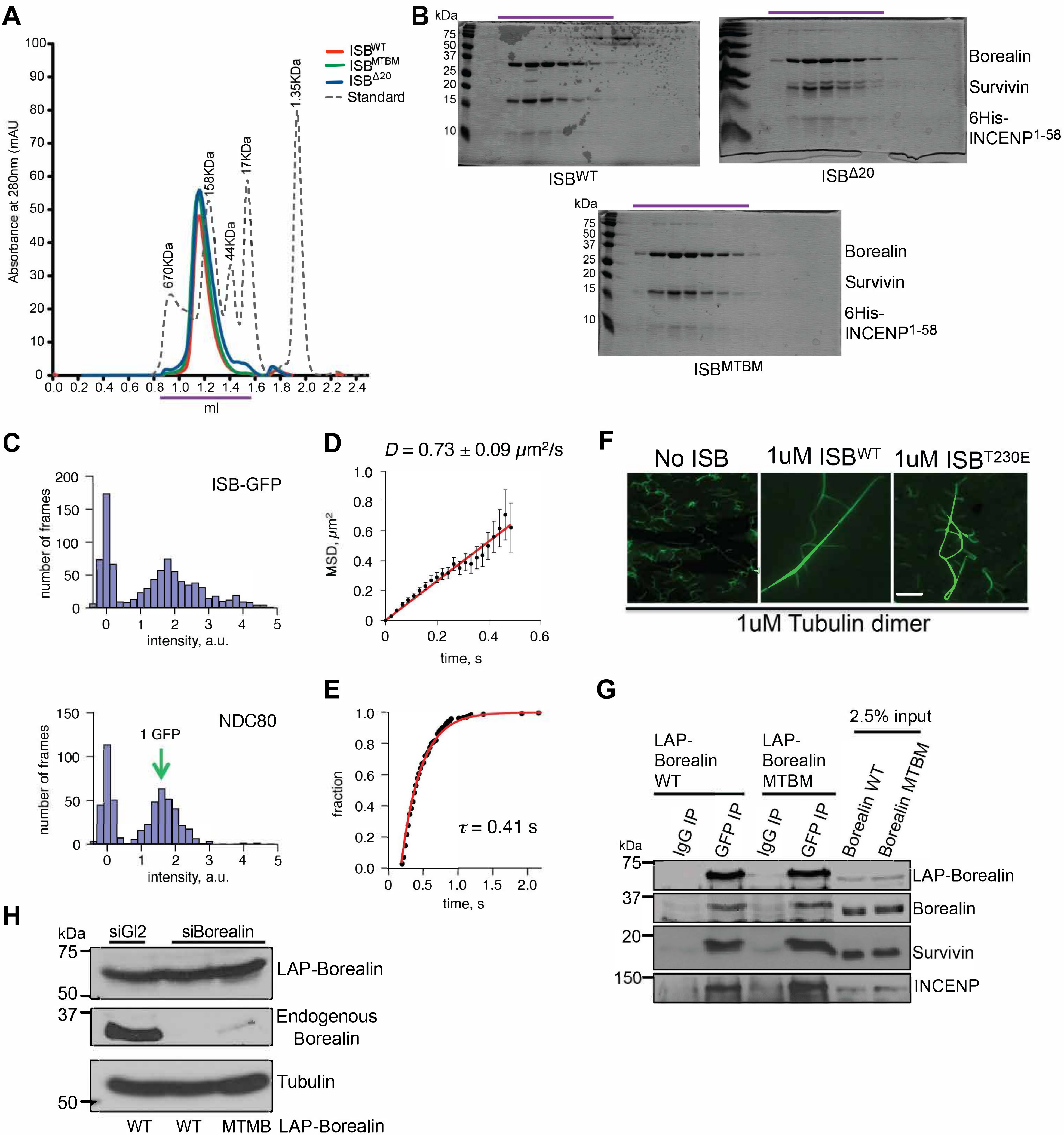

Supplementary Figure 1: The microtubule binding mutations of Borealin have no effect on the ISB or the CPC complex formation. (A) Graph of the FPLC gel-filtration run on a superdex S-200 column. ISB^{WT} (red), ISB^{MTBM} (green), $ISB^{\Delta 20}$ (Blue) and FPLC standard (dashed line). (B) Coomassie stained gel of the fractions from the gel filtration run shown in A, purple line on the graph in A and on the coomassie stained gel images indicates the fractions from the FPLC gel-filtration that were loaded on the gel. (C) Histogram distribution of brightness of individual tracks from experiment. Peak intensity of the ISB-GFP signals coincides with that for the NDC80 complex, suggesting that ISB is predominantly monomeric at this low concentration. (D) Mean squared displacement (MSD) vs. time based on 150 tracks obtained in 2 independent experiments. Displacement coordinates were binned every 22 ms; black dots show mean displacements with SEM. Red line shows linear fit. Data from experiment shown in Fig.1C (E) Cumulative distribution for residency times (based on 189 tracks), data were fitted with truncated exponential distribution (red curve, cut-off 0.16 s). Data from experiment shown in Fig.1C. (F) Images from microtubule bundling assay. ISB^{WT} and ISB^{T230E} were incubated with 1uM taxol stabilized microtubules and probed with anti-tubulin antibody. (G) Western blots from the immuno-precipitation experiment is shown. LAP-$Borealin^{WT}$ and LAP-$Borealin^{MTBM}$ were immuno-precipitated from the nocodazole arrested cell lysate using an anti-GFP antibody. The western blot was probed with antibodies raised against GFP, Borealin, survivin and INCENP. (H) Western blot of the whole cell lysate form the Borealin knockdown rescue experiments as described in Fig.2A. Blots were stained with anti-Borealin antibody and GFP antibody; tubulin staining was used as a loading control.

Supplementary Figure 2

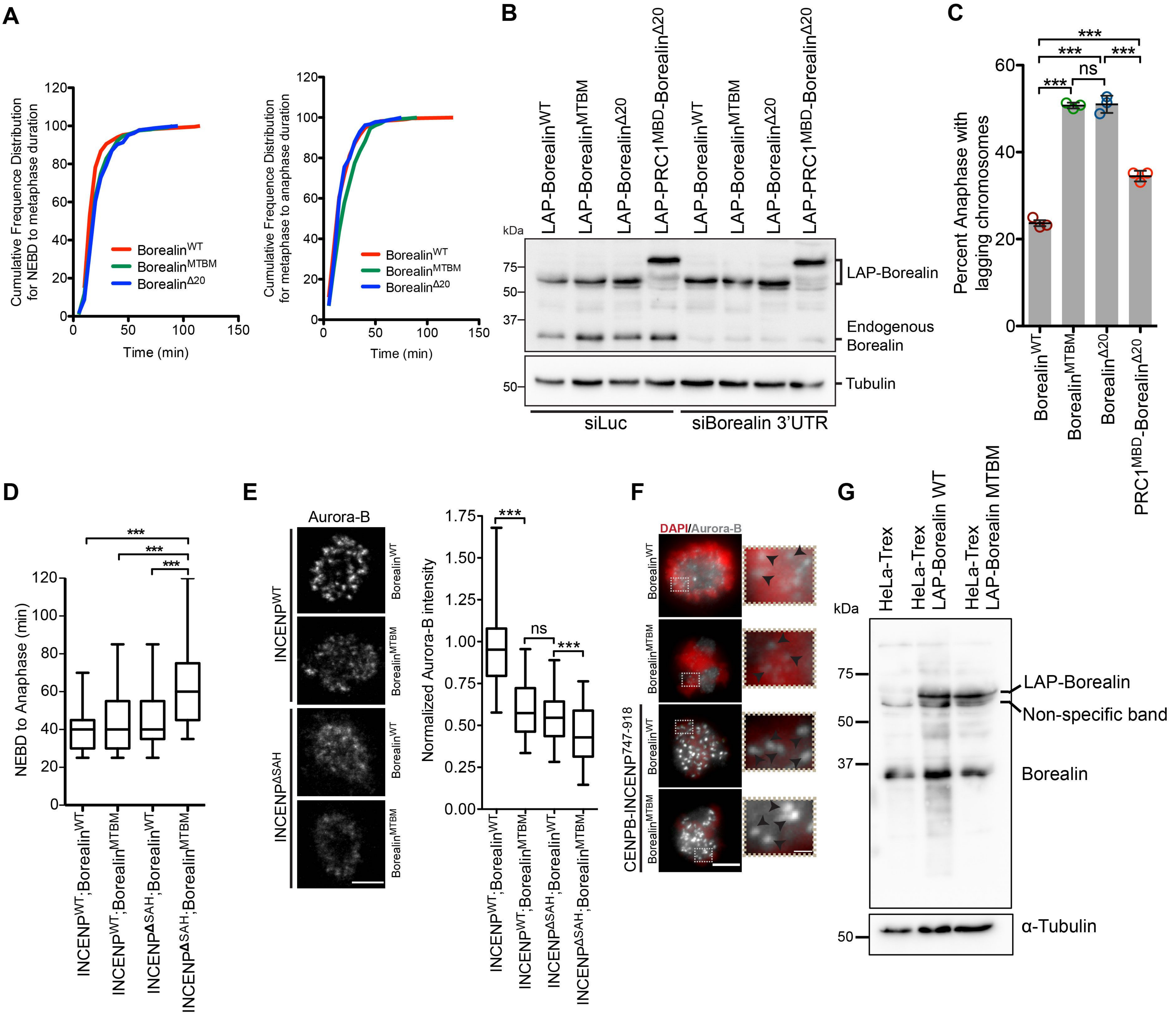

Supplementary Figure 2: Both Borealin and INCENP microtubule binding domain are important for error free mitosis. (A) Cumulative frequency graph for duration of NEBD to metaphase (left) or metaphase to anaphase (right) from data shown in Fig.2D. (B) Western blots of the cells expressing the indicated transgenes and treated with siRNA as in Fig.2A. (C) Bar graph showing percent of cells undergoing anaphase with lagging chromosomes after STLC release assay, cells were treated as described earlier in Fig.2A and rescued with indicated Borealin transgenes. STLC was added 6 hours post 2nd thymidine release for 2hours. STLC was then washed out and 2 hours post washout cells were fixed (data from 3 independent experiments, at least 84 cells were analyzed per experiment). Open circle indicates the individual data points. Error bars represent ±s.d. Statistical analysis was done using one-way ANOVA and Bonferroni's multiple comparison test for both D and E. *** $P<0.001$; ** $P<0.01$, * $P<0.05$. (D) Box and whisker graph of nuclear envelope breakdown (NEBD) to anaphase duration for experiment shown in Fig.2E. Statistical analysis was performed using Dunn's Multiple Comparison Test *** $P<0.001$. (E) HeLa-TReX cells expressing mcherry-Borealin WT or MTBM and INCENP WT or ΔSAH-GFP were treated with Borealin and INCENP 3'UTR siRNA as described in Fig.2A, and immunostained with Aurora-B antibody (Left). Box and whisker graph of normalized Aurora-B intensity (Right). Data is from one of at least 2 replicates. For statistical analysis two-tailed Mann Whitney test was applied (data from at least 7 cells per condition). *** $P<0.0001$, ns $P=0.0580$. (F) HeLa-TReX cells expressing indicated exogenous proteins were treated with Borealin 3'UTR siRNA as described in Fig.2A and 100nM paclitaxel for 1 hour before fixation, and immuno-stained with Aurora-B antibody. Grey dashed boxes represent the area enlarged on the right. Arrowhead points to the inner-centromeric Aurora-B localization. (G) Western blot of HeLa TReX parental cells or cells expressing LAP-Borealin WT or MTBM stained with Borealin

antibody, showing the presence of non-specific band below the LAP-Borealin band (often seen with some batches of Borealin antibody). Tubulin staining was used as a loading control. All Box and whisker plots represent the median (central line), 25th-75th percentile (bounds of the box) and 5th-95th percentile (whiskers). Scale bar is 5um.

Supplementary Figure 3

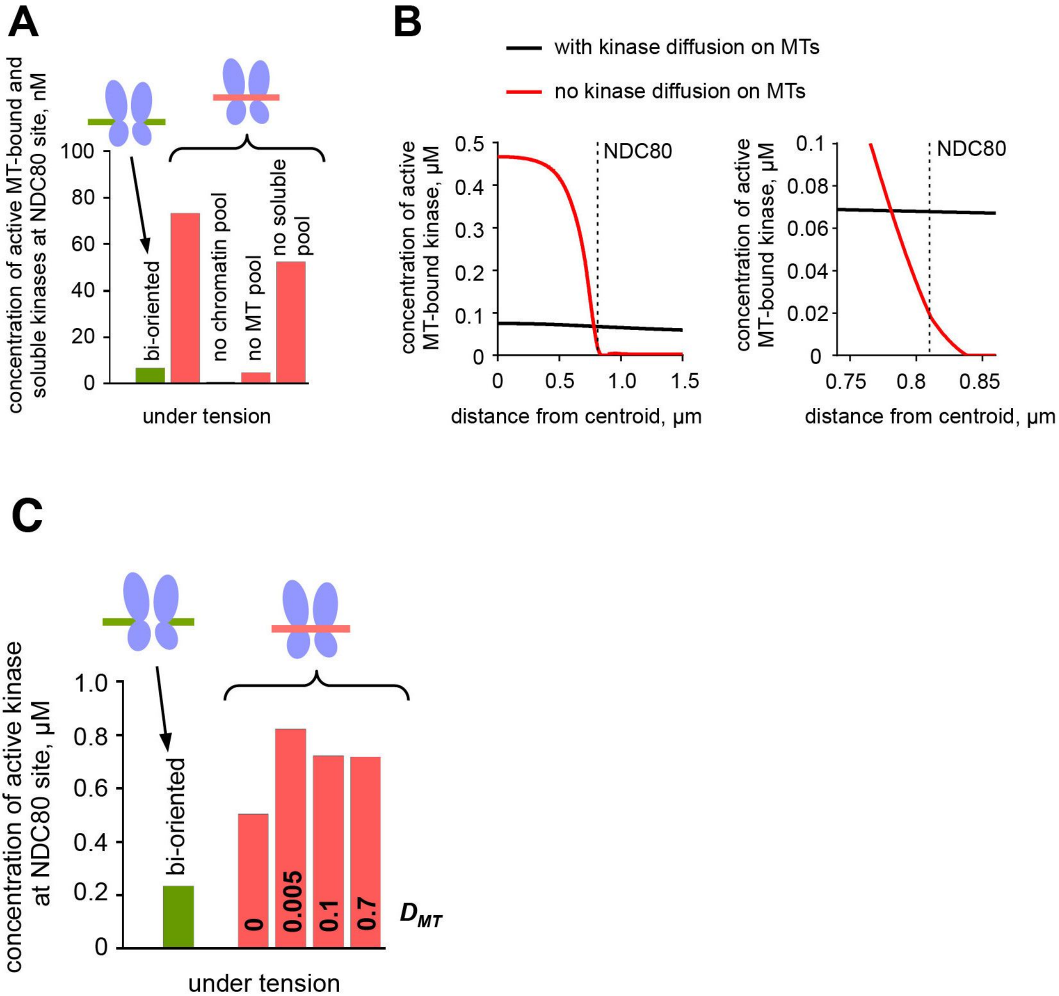

Supplementary Figure 3: Modeling results for spatial distribution of different kinase forms. (A) Same graph as in Fig.4G but plotting the combined activity of only microtubule-bound and soluble pools of kinase. (B) Concentration profiles of active microtubule-bound kinase at the kinetochore with centromere-proximal microtubules. Black curve shows prediction of the main model with CPC diffusion coefficient 0.1 $\mu m^2/s$, showing a gentle gradient. Red curve is prediction for the non-diffusing CPC ($D_{MT} = 0$), showing that in this case the microtubule-bound kinase is significantly more active at the centromere, where it becomes “ignited” by the chromatin-bound kinase. Right graph is an enlargement, showing that in the absence of diffusion, significantly smaller fraction of active CPC can reach the NDC80 site. (C) Quantification of the effect of diffusion of microtubule-bound kinase on the concentration of active kinase (all pools) at NDC80 site at the kinetochore with centromere-proximal microtubules.

Supplementary Figure 4

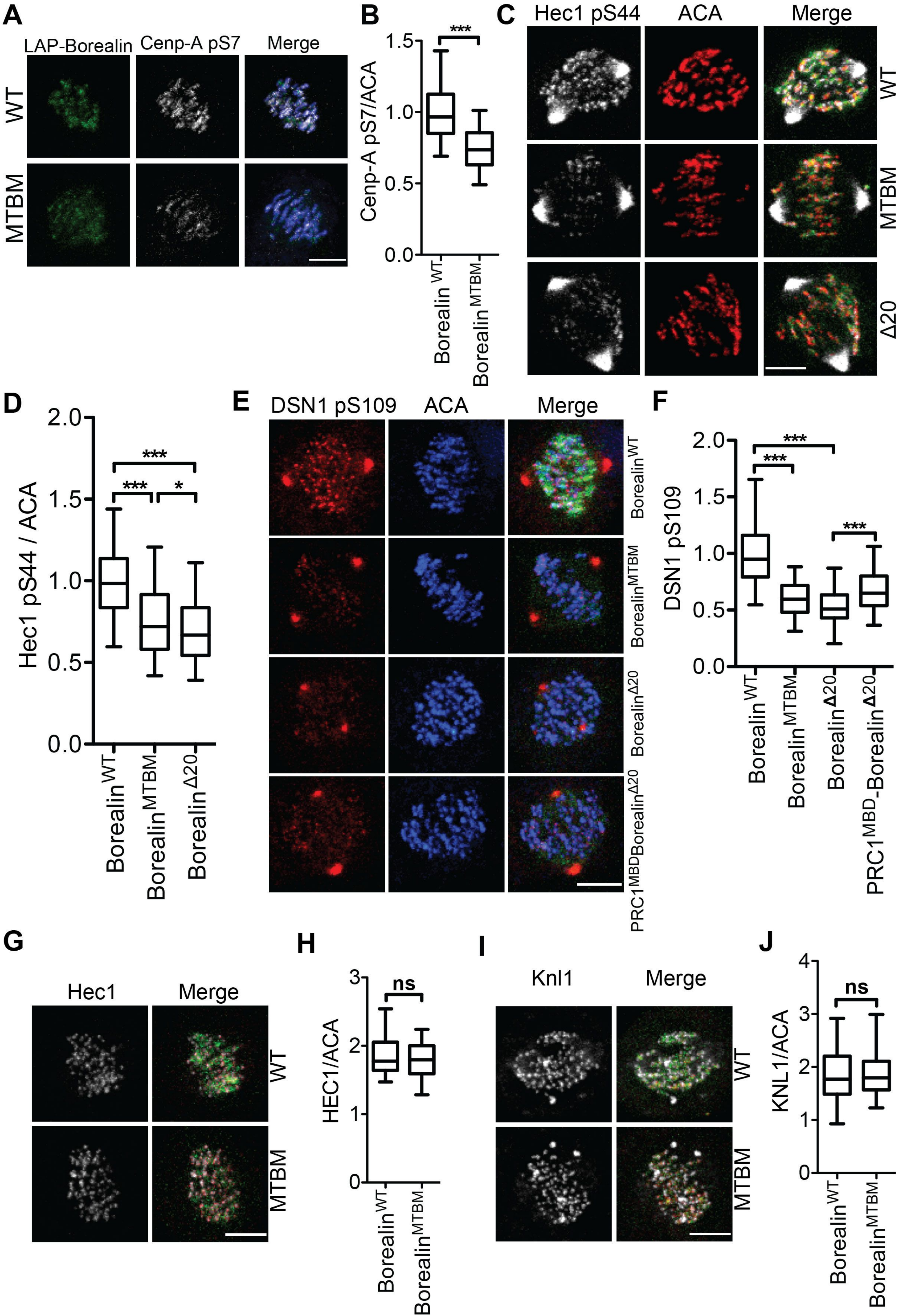

Supplementary Figure 4: Borealin-microtubule interaction is important for robust kinetochore phosphorylation by the CPC. (A) Cells expressing LAP-Borealin^{WT} and LAP-Borealin^{MTBM} were treated as shown in Fig.5A and immunostained for CenpA pS7; representative images are shown. (B) Box and whisker graph of normalized CenpA pS7 intensity (data from at least 8 cells per condition). Statistical analysis was performed using Mann Whitney Test, *** P< 0.0001. (C) Cells expressing LAP-Borealin^{WT}, LAP-Borealin^{MTBM} and LAP-Borealin$^{\Delta 20}$ were treated as described in Fig.5A and immunostained with Hec1 pS44. Representative images are shown. Merge image shows LAP-Borealin (green), ACA (Blue) and Hec1pS44 (grey). (D) Box and whisker graph of the normalized Hec1 pS44 intensity from C (data from at least 10 cells per condition). (E) Cells expressing LAP-Borealin^{WT}, LAP-Borealin^{MTBM}, LAP-Borealin$^{\Delta 20}$ and LAP-PRC1^{MBD}-Borealin$^{\Delta 20}$ were treated with Borealin siRNA as described in Fig.5A. Cells were then immunostained with DSN1 pS109 and ACA. Representative images of the experiment are shown. Merge image shows LAP-Borealin (green), ACA (Blue) and DSN1pS109 (red). (F) Box and whisker graph of the normalized DSN1 pS109 intensity from E (data from at least 8 cells). For D and F statistical analysis was done using one way ANOVA (Kruskal-Wallis test) with Dunn's Multiple Comparison Test, *** P<0.001; ** P<0.01, * P<0.05 and ns P>0.05. Cells were treated as described in Fig.5A and immunostained with Hec1 (G) or Knl1 (I); representative images are shown. Merge image shows LAP-Borealin (green), ACA (red) and Hec1 or Knl1 (grey). Box and whisker graph of normalized Hec1 (H) or Knl1 (J) intensity (data from at least 6 cells per condition). Statistical analysis was performed using Mann Whitney test for H (P=0.3131) and J (P=0.4431). Immunostaining data is from one of at least 2 replicates. All Box and whisker plots represent the median (central line), 25th-75th percentile (bounds of the box) and 5th-95th percentile (whiskers). Scale bar 5um.

Supplementary Figure 5

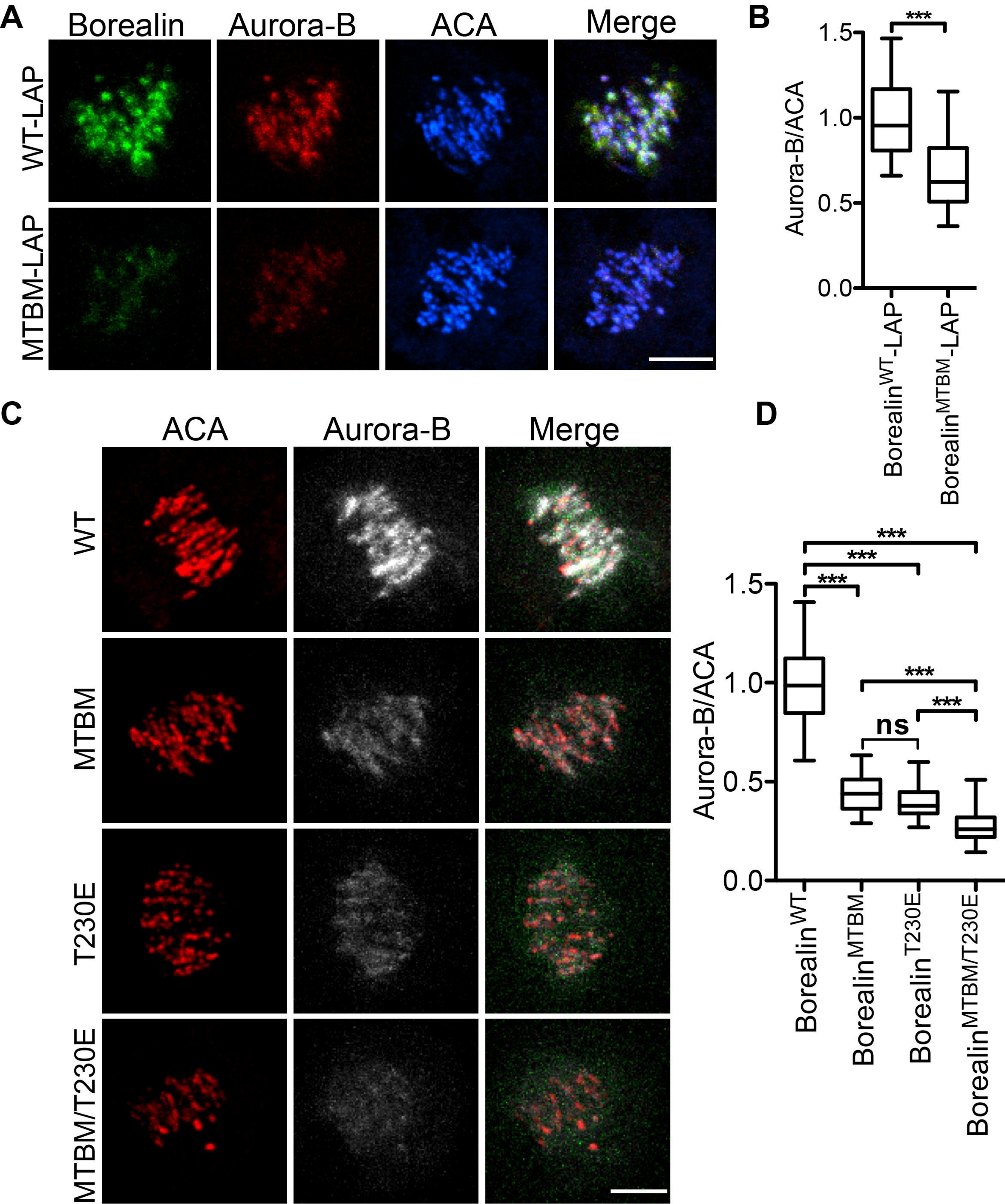

Supplementary Figure 5: Borealin microtubule-binding domain and dimerization domain independently enhance inner-centromere localization of the CPC. (A) Cells expressing C-terminal LAP tagged Borealin^{WT} or Borealin^{MTBM} were treated as in Fig.5A and immunostained with antibodies against Aurora-B and ACA. Representative images from the experiments are shown. (B) Box and whisker graph of the normalized Aurora-B intensity from A (data from at least 5 cells per condition). Statistical analysis was performed using Mann Whitney Test, *** P< 0.0001. (C) Representative images are shown from the experiment were cells expressing LAP-Borealin^{WT}, LAP-Borealin^{MTBM}, LAP-Borealin^{T230E} (Borealin dimerization mutant) and LAP-Borealin$^{MTBM/T230E}$ were treated as in Fig.5A and immunostained with antibodies against Aurora-B and ACA. (D) Box and whisker graph of the normalized Aurora-B intensity from C (data from at least 7 cells per condition). Statistical analysis was done using one way ANOVA (Kruskal-Wallis test) with Dunn's Multiple Comparison Test, *** P<0.001; ** P<0.01, * P<0.05 and ns P>0.05. Immunostaining data is from one of at least 2 replicates. All Box and whisker plots represent the median (central line), 25th-75th percentile (bounds of the box) and 5th-95th percentile (whiskers). Scale bar is 5um.

Supplementary Figure 6

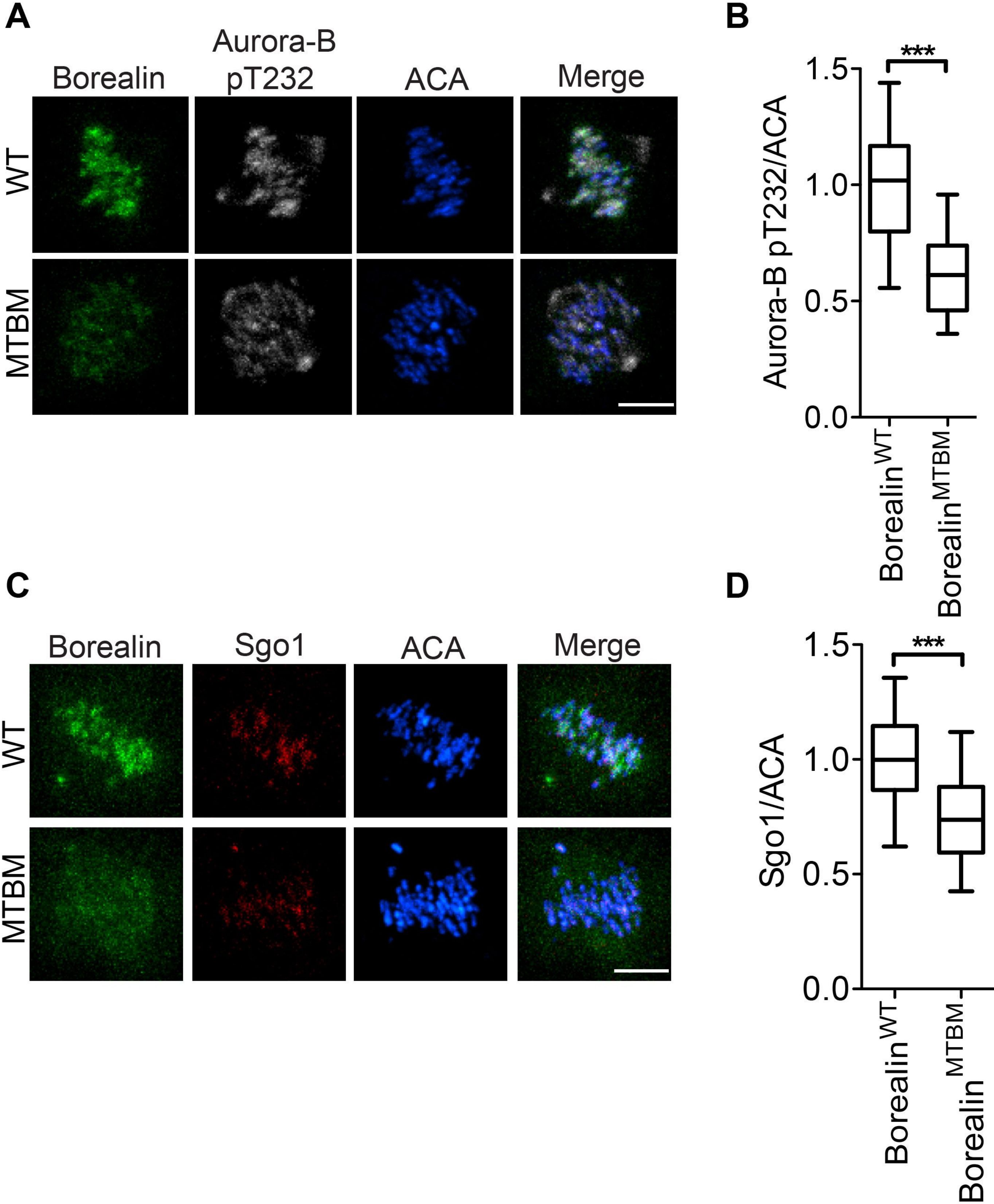

Supplementary Figure 6: Borealin-microtubule interaction is important for proper centromeric Sgo1 localization. Representative images are shown from the experiment were cells expressing LAP-Borealin^{WT} and LAP-Borealin^{MTBM} were treated as in Fig.5A and immunostained with Aurora-B pT232 (A), Sgo1 (C) and ACA. Representative images are shown in A and C. Box and whisker graph of normalized Aurora-B pT232 intensity (B) (data from at least 10 cells) and normalized Sgo1 intensity (D) (data from at least 8 cells per condition) in cells expressing LAP-Borealin^{WT} and LAP-Borealin^{MTBM}. Statistical analysis was performed using Mann Whitney Test, *** P< 0.0001. Immunostaining data is from one of at least 2 replicates All Box and whisker plots represent the median (central line), 25th-75th percentile (bounds of the box) and 5th-95th percentile (whiskers). Scale bar is 5um.

Supplementary Figure 7

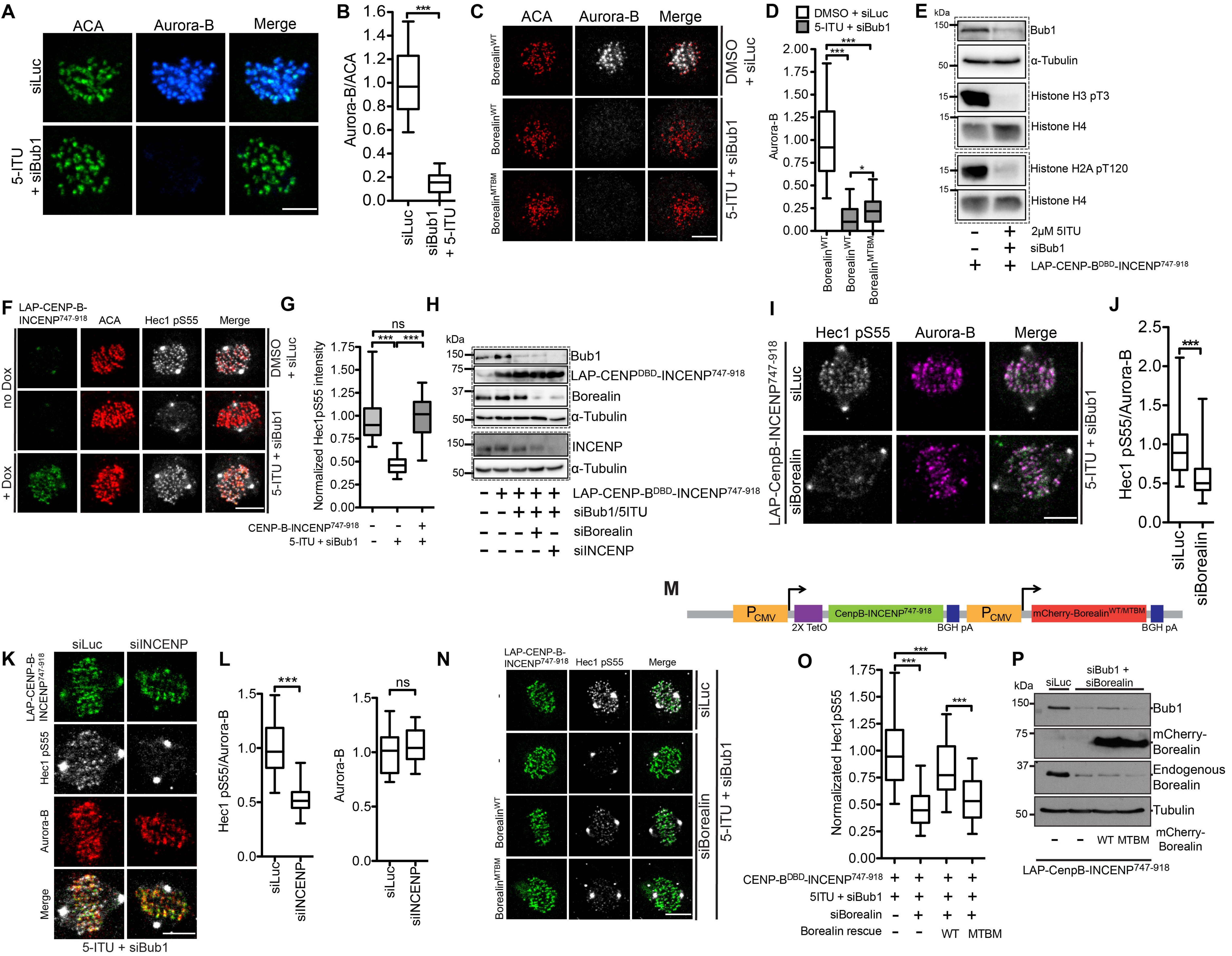

Supplementary Figure 7: Borealin mediated non-centromeric CPC-microtubule interaction is required for robust phosphorylation of the kinetochore substrates by the CPC. (A) HeLa-Trex cells were either treated as in Fig.8A (siBub1 and 2uM 5-ITU), with the exception of doxycycline treatment, or untreated (control). Cells were stained for Aurora-B and ACA after treatment with 3.3uM Nocodazole. (B) Box and whisker graph of normalized Aurora-B intensity from experiment shown in A (data from at least 8 cells per condition). Statistical analysis was done using Mann Whitney Test, *** $P< 0.0001$. Scale bar 5um. (C) HeLa TReX cells expressing the indicated transgene were treated as in supplement Fig.7A with addition of Borealin 3'UTR siRNA treatment. The cells were then stained for Aurora-B (grey) and ACA (red). (D) Box and whisker plot of the Aurora-B intensity from experiment shown in C (combined data from at least 8 cells per condition). Statistical analysis was performed using one-way ANOVA and Bonferroni's Multiple Comparison Test. *** $P<0.001$; * $P<0.05$. (E) Western blot of HeLa TReX lysates under indicated condition stained with indicated antibodies. All the blots inside the dotted box indicates antibodies probed on the same membrane, the blots in two different dotted box are the same lysates run parallel on different lanes. (F) Cells were treated as described in Fig.8A in indicated conditions followed by immunostaining with Hec1 pS55 and ACA antibodies. Representative images are shown. (G) Box and whisker graph of normalized Hec1 pS55 from experiment shown in F (combined data from at least 8 cells per condition). Statistical analysis was performed using one-way ANOVA and Bonferroni's Multiple Comparison Test. *** $P<0.001$; * $P<0.05$, ns $P>0.05$. (H) Western blot of HeLa TReX lysates under indicated condition stained with indicated antibodies. All the blots inside the dotted box indicates antibodies probed on the same membrane, the blots in two different dotted box are the same lysates run parallel on different lanes. (I) Cells were treated as described in Fig.8A followed by immunostaining

with Hec1 pS55 and Aurora-B antibody. Representative images are shown. (J) Box and whisker graph of normalized Hec1 pS55 from experiment shown in I (data from at least 9 cells per condition). Statistical analysis was done using Mann Whitney Test, *** P< 0.0001. (K) Cells were treated as described in Fig.8A, instead of borealin siRNA INCENP siRNA was used, followed by immunostaining with Hec1 pS55 and Aurora-B antibodies. Representative images are shown. (L) Box and whisker graph of normalized Hec1 pS55 from experiment shown in K (combined data from at least 10 cells per condition). Statistical analysis was done using Mann Whitney Test, *** P< 0.0001, ns P=0.0628. (M) Schematic of the construct used for making cell lines stably expressing LAP-CenpB^{DBD}-INCENP$^{747-918}$ under doxycycline induction and constitutively expressing mcherry-Borealin$^{WT\ or\ MTBM}$. (N) Cells were treated as described in Fig.8E followed by immunostaining with Hec1 pS55. Representative images are shown. (O) Box and whisker graph of normalized Hec1 pS55 from experiment shown in N (combined data from at least 7 cells per condition). Statistical analysis was performed using one-way ANOVA and Bonferroni's Multiple Comparison Test. *** P<0.001. (H) Western blot showing Borealin and Bub1 knockdown and expression of mCherry-Borealin$^{WT\ or\ MTBM}$ transgene in cells expressing LAP-CenpB-INCENP$^{747-918}$. Tubulin blot is used as loading control. Immunostaining data is from one of at least 2 replicates. All Box and whisker plots represent the median (central line), 25th-75th percentile (bounds of the box) and 5th-95th percentile (whiskers). Scale bar is 5um for all the images except for images in K where it is 4um.

Supplementary Figure 8

Fig1A

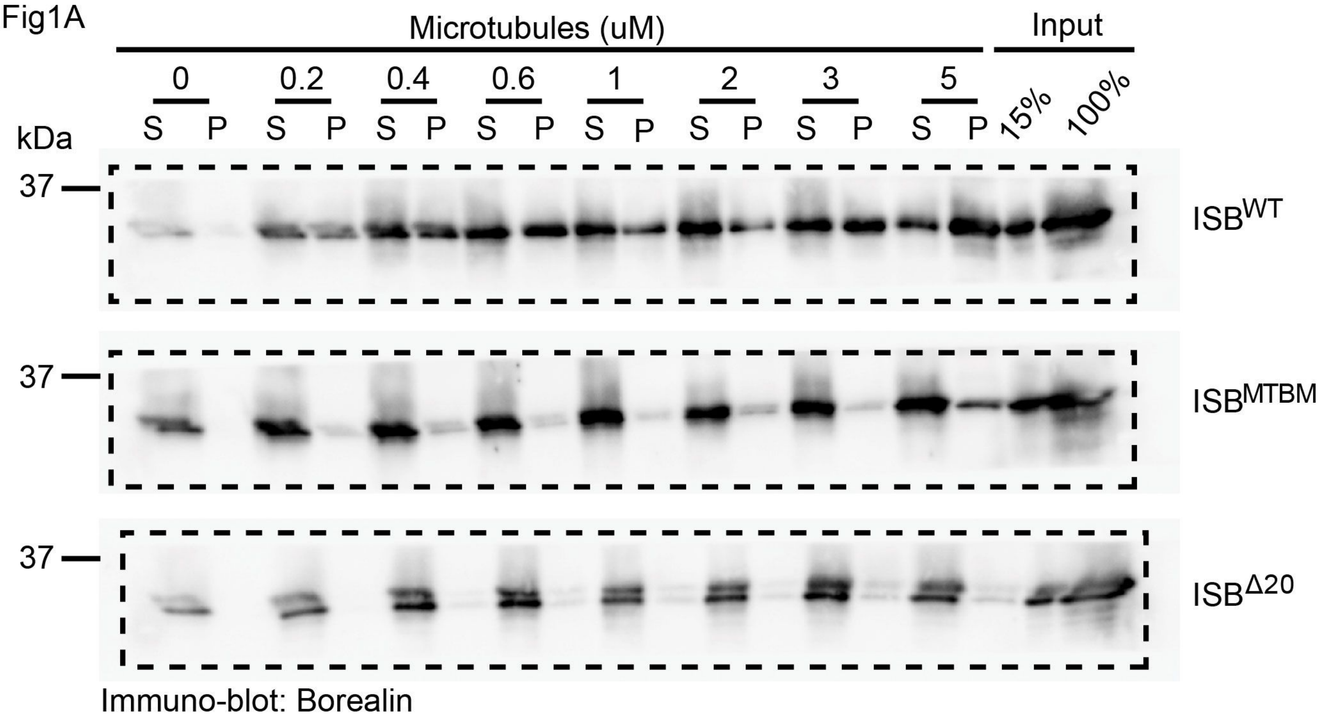

Supplement Fig 1G

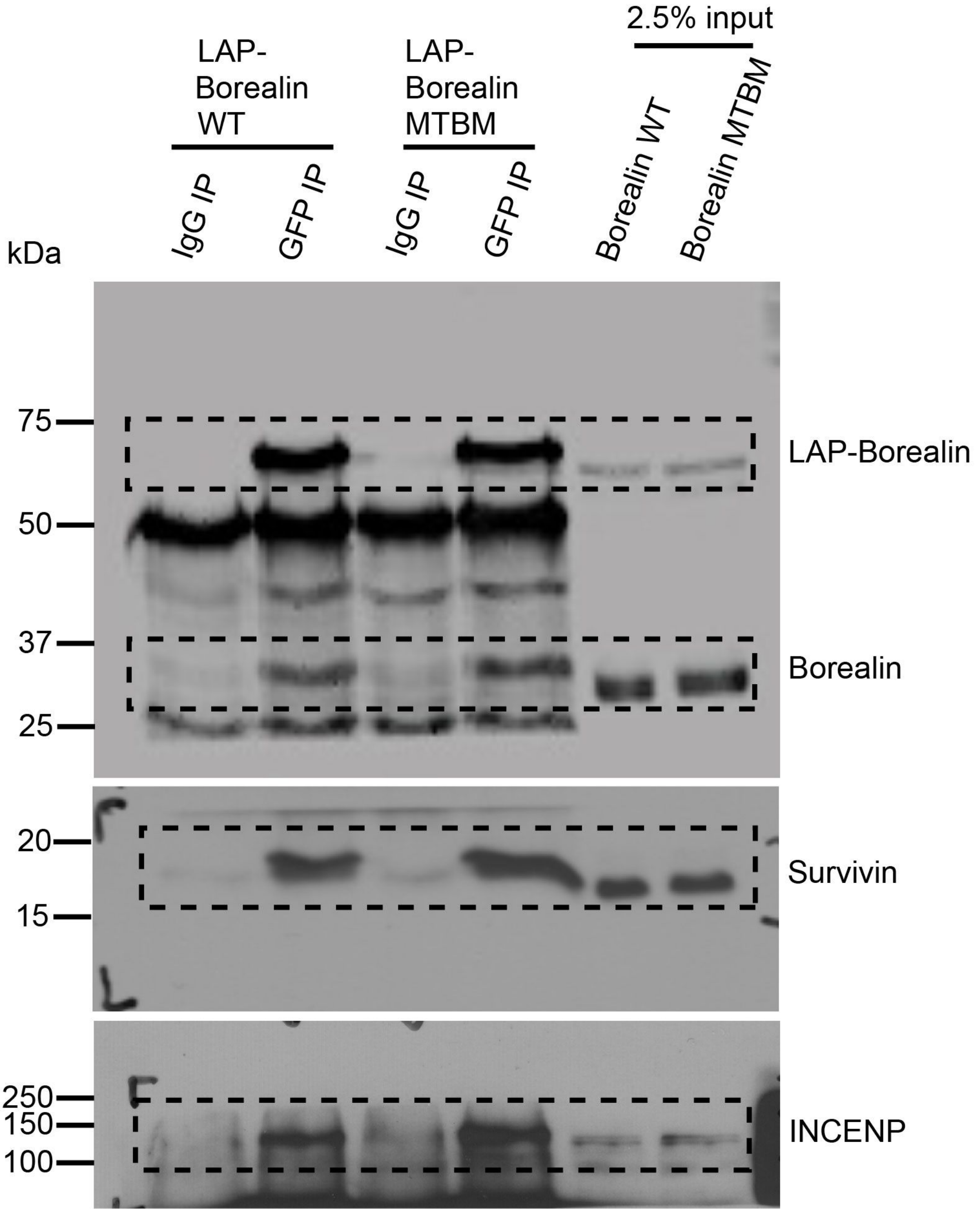

Supplement Fig 1H

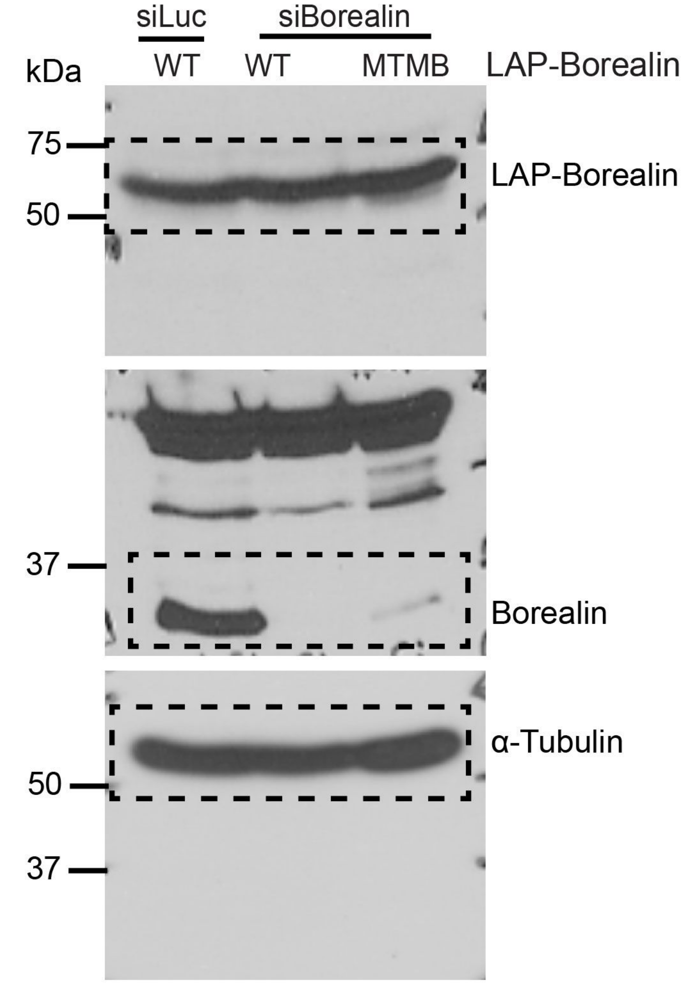

Supplementary Figure 8: Uncropped scans of blots from Fig.1A, Supplement Fig.1G and H.

Supplementary Figure 9

Fig 2C

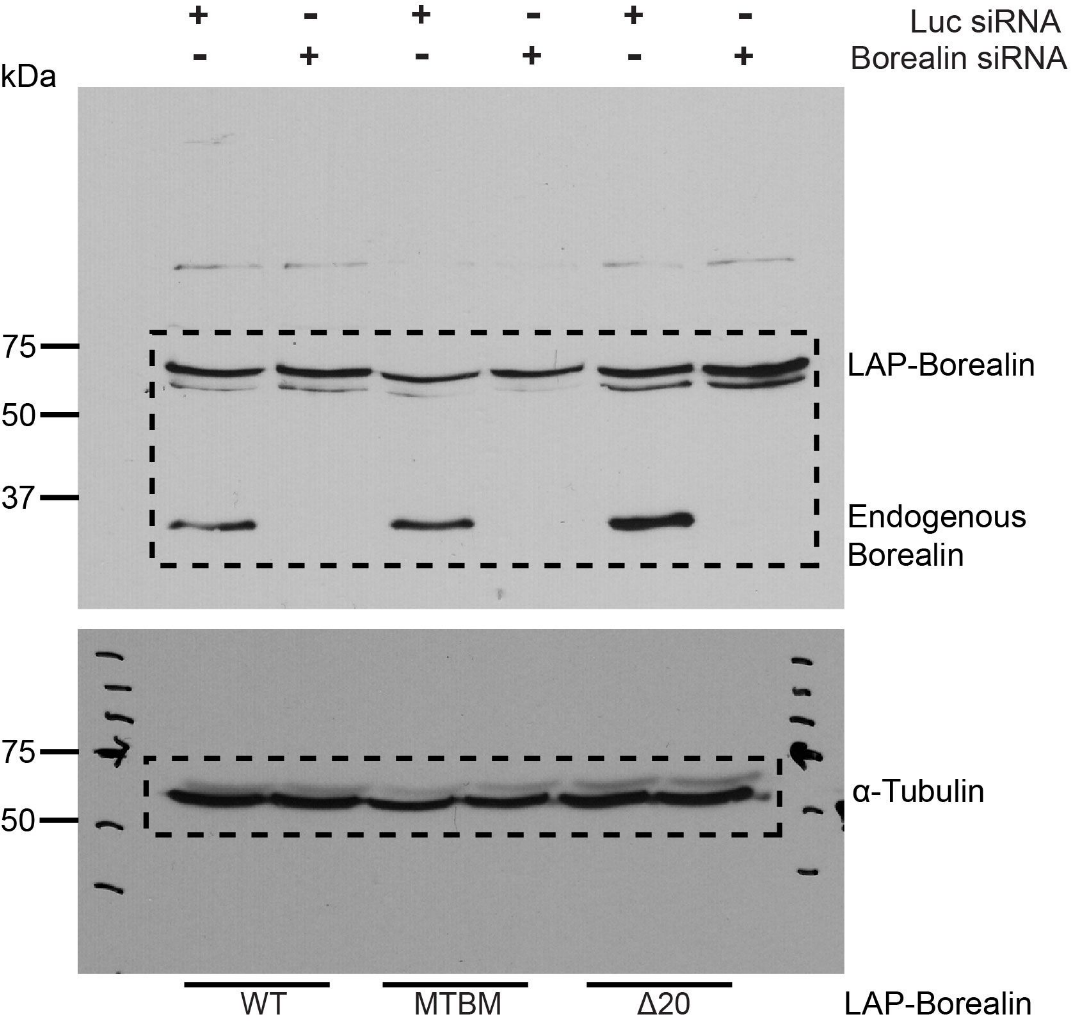

Supplement Fig 2B

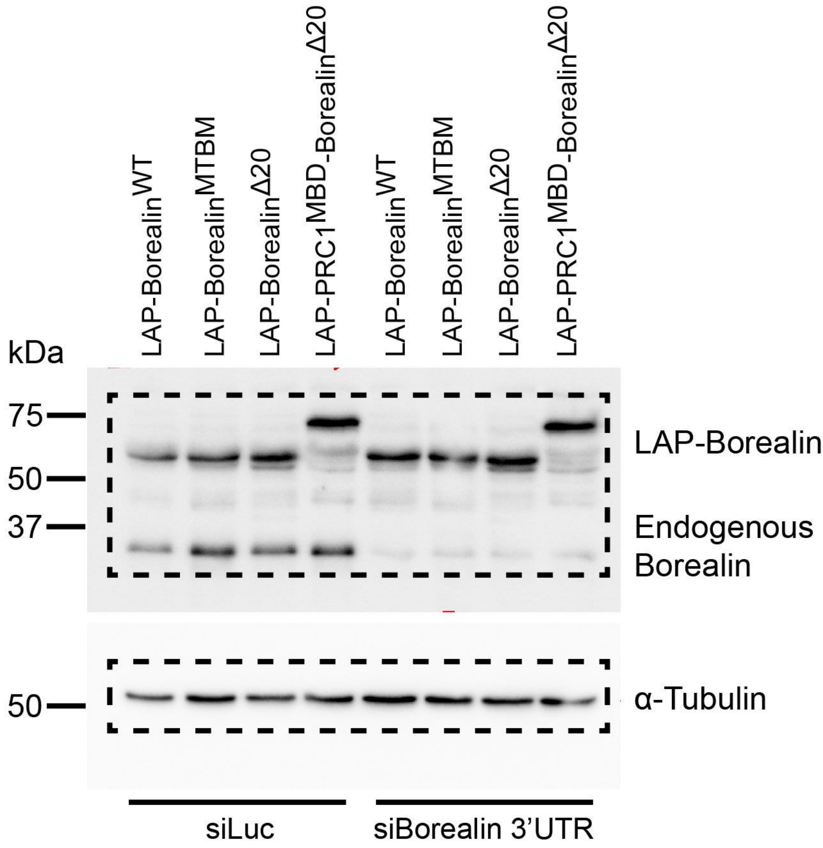

Supplementary Figure 9: Uncropped scans of blots from Fig.2C, Supplement Fig.2B.

Supplementary Figure 10

Supplement Fig 7E

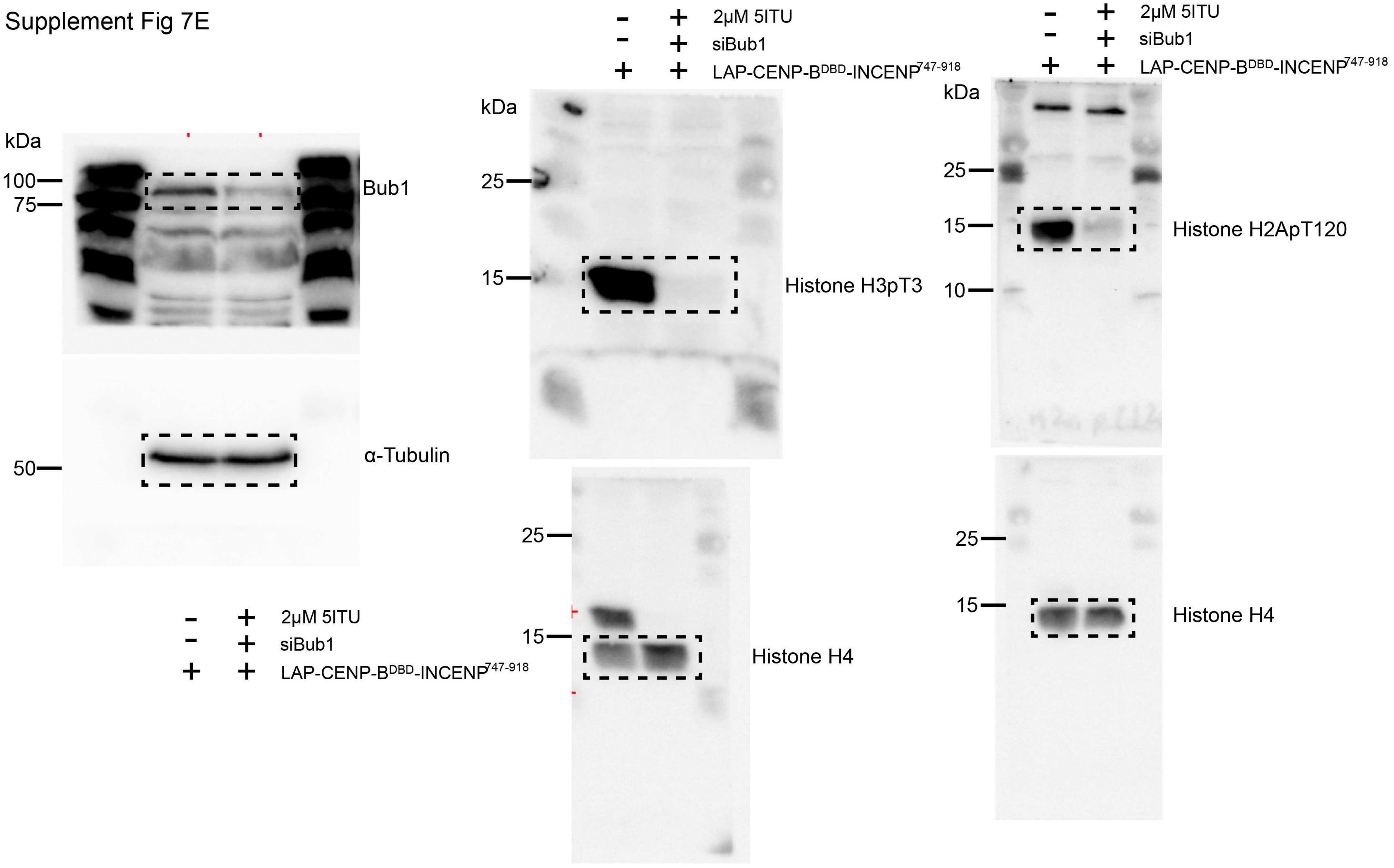

Supplement Fig 7H

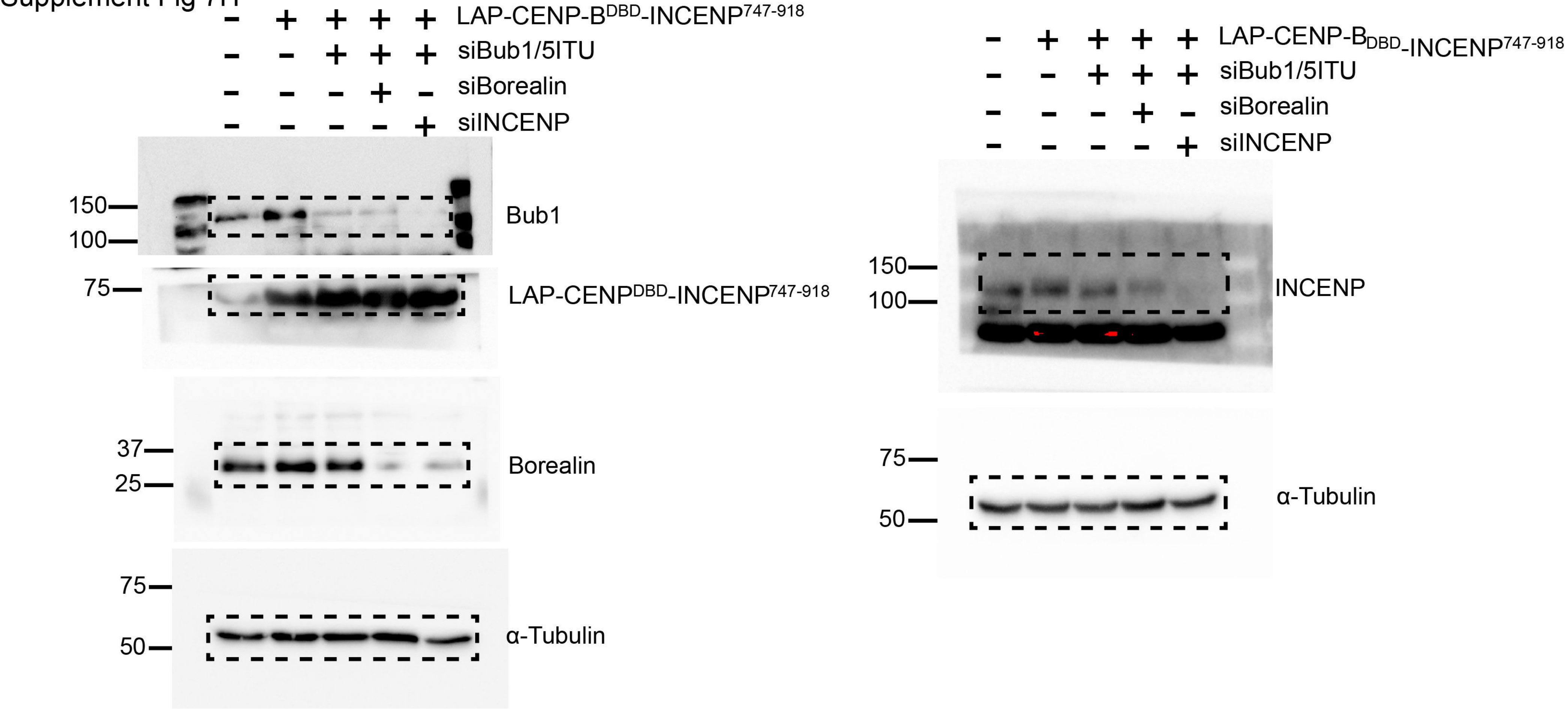

Supplement Fig 7P

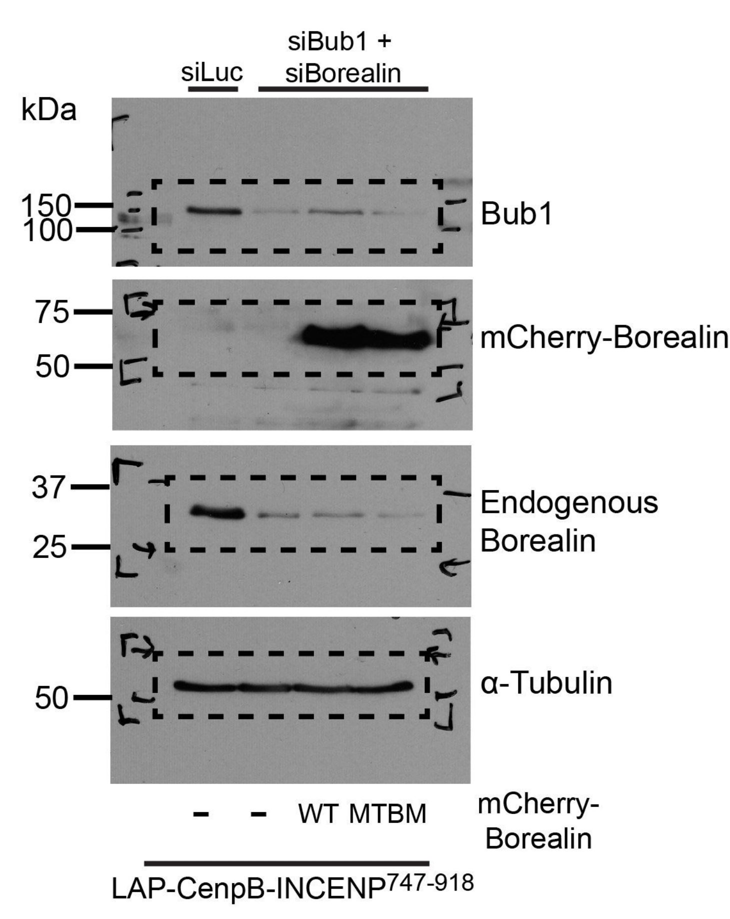

Supplementary Figure 10: Uncropped scans of blots from Supplement Fig.7E, 7H and 7P.

Supplementary References

67. Cheeseman, I. M., Hori, T., Fukagawa, T. & Desai, A. KNL1 and the CENP-H/ I/K complex coordinately direct kinetochore assembly in vertebrates. Mol. Biol. Cell 19, 587–594 (2008).

68. Klein, U. R., Nigg, E. A. & Gruneberg, U. Centromere targeting of the chromosomal passenger complex requires a ternary subcomplex of borealin, survivin, and the N-terminal domain of INCENP. Mol. Biol. Cell 17, 2547–2558 (2006).

69. Jia, L., Li, B. & Yu, H. The Bub1-Plk1 kinase complex promotes spindle checkpoint signalling through Cdc20 phosphorylation. Nat. Commun. 7, 10818 (2016).
