# Supplementary material for: The binding of Borealin to microtubules underlies a tension independent kinetochore-microtubule error correction pathway
Source: Nat Commun. 2019 Feb 8;10:682. doi: 10.1038/s41467-019-08418-4 (PMC6368601; doi:10.1038/s41467-019-08418-4)
Supplement: Supplementary file 3 — Reporting Summary [file 41467_2019_8418_MOESM3_ESM.pdf]

nature research

Corresponding author(s): P.Todd Stukenberg
Ekaterina Grishchuk

Reporting Summary

Nature Research wishes to improve the reproducibility of the work that we publish. This form provides structure for consistency and transparency in reporting. For further information on Nature Research policies, see Authors & Referees and the Editorial Policy Checklist.

Statistical parameters

When statistical analyses are reported, confirm that the following items are present in the relevant location (e.g. figure legend, table legend, main text, or Methods section).

n/a	Confirmed	
☐	☒	The exact sample size (*n*) for each experimental group/condition, given as a discrete number and unit of measurement
☐	☒	An indication of whether measurements were taken from distinct samples or whether the same sample was measured repeatedly
☐	☒	The statistical test(s) used AND whether they are one- or two-sided<br>*Only common tests should be described solely by name; describe more complex techniques in the Methods section.*
☐	☒	A description of all covariates tested
☐	☒	A description of any assumptions or corrections, such as tests of normality and adjustment for multiple comparisons
☐	☒	A full description of the statistics including central tendency (e.g. means) or other basic estimates (e.g. regression coefficient) AND variation (e.g. standard deviation) or associated estimates of uncertainty (e.g. confidence intervals)
☐	☒	For null hypothesis testing, the test statistic (e.g. *F*, *t*, *r*) with confidence intervals, effect sizes, degrees of freedom and *P* value noted<br>*Give P values as exact values whenever suitable.*
☒	☐	For Bayesian analysis, information on the choice of priors and Markov chain Monte Carlo settings
☒	☐	For hierarchical and complex designs, identification of the appropriate level for tests and full reporting of outcomes
☒	☐	Estimates of effect sizes (e.g. Cohen's *d*, Pearson's *r*), indicating how they were calculated
☐	☒	Clearly defined error bars<br>*State explicitly what error bars represent (e.g. SD, SE, CI)*

Our web collection on statistics for biologists may be useful.

Software and code

Policy information about availability of computer code

Data collection	Software are indicated at relevant location. We have used Volocity (Perkin Elmer).
Data analysis	Software are indicated at relevant location. We have used Volocity, Prism (Graphpad), Mathematica.

For manuscripts utilizing custom algorithms or software that are central to the research but not yet described in published literature, software must be made available to editors/reviewers upon request. We strongly encourage code deposition in a community repository (e.g. GitHub). See the Nature Research guidelines for submitting code & software for further information.

Data

Policy information about availability of data

All manuscripts must include a data availability statement. This statement should provide the following information, where applicable:

- Accession codes, unique identifiers, or web links for publicly available datasets
- A list of figures that have associated raw data
- A description of any restrictions on data availability

All relevant data will be available upon request from the authors.

Field-specific reporting

Please select the best fit for your research. If you are not sure, read the appropriate sections before making your selection.

☒ Life sciences ☐ Behavioural & social sciences ☐ Ecological, evolutionary & environmental sciences

For a reference copy of the document with all sections, see nature.com/authors/policies/ReportingSummary-flat.pdf

Life sciences study design

All studies must disclose on these points even when the disclosure is negative.

Sample size	Sample size were chosen based on prior knowledge for obtaining significant p-values from the type of experiment performed.
Data exclusions	No data were excluded
Replication	All experiments were replicated at least twice and often more times as mentioned in the mauscript
Randomization	Cells were randomly selected for imaging.
Blinding	no blinding was performed

Reporting for specific materials, systems and methods

Materials & experimental systems

n/a	Involved in the study	
☒	☐	Unique biological materials
☐	☒	Antibodies
☐	☒	Eukaryotic cell lines
☒	☐	Palaeontology
☒	☐	Animals and other organisms
☒	☐	Human research participants

Methods

n/a	Involved in the study	
☒	☐	ChIP-seq
☒	☐	Flow cytometry
☒	☐	MRI-based neuroimaging

Antibodies

Antibodies used	Mouse monoclonal Aurora-B antibody (BD bisciences, Cat.no. 611083); Rabbit polyclonall Borealin antibody (Stukenberg lab); Mouse monoclonal INCENP antibody (Abcam, cat.no. ab23956); ACA antibody (Antibodies inc, Cat.no. 15-234-0001); Rabbit polyclonal CENP-T antibody (Foltz lab); Rb polyclonal pH3S10 antibody (EMD Millipore, Cat.no. 06-570), Rb polyclonal pH3T3 antibody (EMD millipore, cat. no. 07-424); Rb polyclonal H2A pT120 antibody (Active motif, Cat. no. 61195); Ms monoclonal Sgo1 antibody (Abcam, Cat. no. ab58023); Rb polyclonal Aur-B pT232 antibody (Rockland, cat. no. 600-401-677S); Rb polyclonal CENPA pS7 (EMD millipore, cat.no. 07-232); Rb polyclonal Dsn1 pS109, Knl1 pS60 (Cheeseman lab); Rb polyclonal Hec1 pS44 (Deluca lab); Rb polyclonal Hec1 pS55 and pS69 antibodies (Stukenberg lab); Ms monoclonal Hec1 antibody (Genetex, cat. no. GTX70268); Rb polyclonal Knl1 antibody (Desai lab); Rb polyclonal Bub1 antibody (Genetex, Cat.no. GTX30097), Rb polyclonal mCherry antibody (Genetex, cat.no.GTX128508); Rb polyclonal INCENP antbody (Sigma, cat.no. I5283); Rb polyclonal Survivin antibody (Cell Signaling, Cat.no. 2808), Ms monoclonal Tubulin antibody (Sigma, T6199).
Validation	We used only previously published and validated or commercial antibodies.

Eukaryotic cell lines

Policy information about cell lines

Cell line source(s)	HeLa-TReX cells were a gift from Dan Foltz Lab.
Authentication	HeLa-TReX cell lines were authenticated to be homogenous population of close derivative on HeLa cells by ATCC.
Mycoplasma contamination	Cell lines were not tested for mycoplasma contamination.

Commonly misidentified lines<br>(See ICLAC register)	No commonly misidentified cell lines were used in this study
